# Localized Multi-Site Knee Bioimpedance as a Predictor for Knee Osteoarthritis Associated Pain Within Older Adults During Free-Living

**DOI:** 10.1109/OJEMB.2023.3256181

**Published:** 2023-03-13

**Authors:** Shelby Critcher, Patricia Parmelee, Todd J. Freeborn

**Affiliations:** ^1^ Department of Electrical and Computer EngineeringThe University of Alabama 8063 Tuscaloosa AL 35487 USA; ^2^ Department of PsychologyThe University of Alabama 8063 Tuscaloosa AL 35487 USA

**Keywords:** Knee Osteoarthritis, Pain, Older-Adults, Bioimpendance, Wearables

## Abstract

The drastic increase in the aging population has increased the prevalence of osteoarthritis in the United States. The ability to monitor symptoms of osteoarthritis (such as pain) within a free-living environment could improve understanding of each person's experiences with this disease and provide opportunities to personalize treatments specific to each person and their experience. In this work, localized knee tissue bioimpedance and self-reports of knee pain were collected from older adults ($N=20$) with and without knee osteoarthritis over 7 days of free-living to evaluate if knee tissue bioimpedance is associated with persons' knee pain experience. Within the group of persons' with knee osteoarthritis increases in 128 kHz per-length resistance and decreases in 40 kHz per-length reactance were associated with increased probability of persons having active knee pain ($p=0.038$ and $p=0.044$).

## I. Introduction

**Fig. 1.**
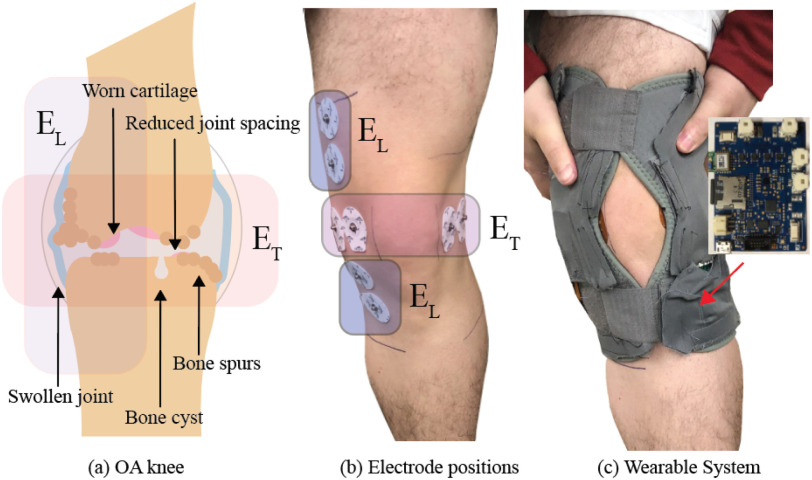
(a) Sample of OA-associated knee joint changes, (b) electrode locations to capture bioimpedance data of knee joint, and (c) wearable electronic sensing system for study data collection.

Osteoarthritis (oa) is a joint disease that can affect the hands, feet, hips, neck, knee and back. Of these joints the knee is the site most commonly affected by OA [1]. This joint disease causes changes that include worn cartilage, bone spurs, reduced joint spacing, and swelling. These knee tissue changes are illustrated in Fig. 1(a). Symptoms of OA can include pain and functional disability which drastically affect the daily lives of those who live with this disease [2]. In addition to its impact, the increase in the aging population within the United States has resulted in an increase in the prevalence of OA [3]. From $2008-2014$ people over the age of 65 accounted for 43% of the total number of people ($>32$ million) with OA in the United States [4]. Within the aging population, many OA symptoms accelerate the functional decline and can lead to increased depression for affected individuals [2]. Studies have reported depression levels three times higher in older adults with OA; additionally feelings of distress are also associated with increased levels of pain and reduced levels of activity [5], [6]. This further highlights that this disease impacts quality of life in terms of both physical abilities and mental health.

Diagnosis and tracking of OA progression is achieved using joint imaging (e.g. x-ray or ultrasound) and self-reports of OA-associated symptoms (e.g. pain, fatigue, depression, mobility) [7], [8]. While imaging techniques can capture changes in joint spacing and hard tissues (e.g. bone), it is limited to late-stage OA progression and requires repeated clinical visits over months and years. This places a significant burden on the patient and is resource intensive in terms of equipment and required medical personnel. While participant self-reports of symptoms do not require expensive equipment when reported as a scaled response during clinical visits, they are still burdensome on the patient and medical personnel. Reducing the burden of tracking OA related symptoms and joint changes has the potential to increase the available data for medical evaluation and provide a path forward to personalize treatments aimed at improving functional abilities and quality of life of persons with OA.

With regard to measuring and reporting physiological data with little burden, wearable devices are a potential solution that are being widely explored. For example, wearable devices have previously been implemented to monitor the knee joint for gait assessment and kinematics of the knee [9] with overall goals to assess knee joint health and to assist in rehabilitation [10], [11], [12]. While these previously implemented sensing technologies can provide information related to the movement profile of the knee, they fail to provide information regarding the underlying physiological changes of the knee [10] and do not capture participant reports of symptoms.

Beyond technologies that can measure knee kinematics one sensing modality, refereed to as bioimpedance spectroscopy (BIS), is being investigated as a sensing technique to accurately measure and characterize localized tissues. Bioimpedance spectroscopy quantifies the passive, frequency-dependent electrical properties of a biological tissue. The electrical properties of a tissue are dependent on tissue type, structure/geometry, and fluid status. Recently BIS has been investigated as a technique to monitor skeletal muscle fatigue [13], [14], segmental fluid shifts [15], blood pressure monitoring [16], [17], knee joint health [18], [19], [20], [21], [22], and for muscle assessment of patients with back pain [23].

Focusing on recent efforts investigating BIS for knee-joint monitoring, Hersek et al. reported injured knees with an ACL or meniscal tear having lower resistance and higher reactance compared to healthy knees [19]. Their results support that this technique is able to capture localized knee-site tissue changes related to injury. Additionally, Ye et al. investigated the differences in impedance metrics from injured and healthy knees, with injury classifications ranging from osteoarthritis to ACL tears [21]. They reported statistically significant relative impedance differences between groups from 46.4 kHz to 1 MHz. Another study also compared bioimpedance measurements before and after a total knee replacement to monitor post surgical swelling [24]. The commonly used bioimpedance metric, $R_{0}$, is associated with the extracellular fluid of tissue and is where most edema occurs. This metric was compared pre- and post- knee replacement surgery with reports of significant differences in $R_{0}$ between baseline and the post-surgery measurements [24]. This further supports bioimpedance measurements are associated with extra-cellular fluid changes of localized tissues. Additionally, a wearable sensing system was investigated by Richardson et al. to quantify rheumatoid arthritis (RA) in the knee [25]. This study reports promising use of BIS measurements along with additional sensing modalities in the detection and monitoring of inflammation.

The results of these studies support that the electrical impedance of knee joint tissues are impacted by injury, fluid accumulation, and arthritis status (e.g. having either OA and RA). However, none of these works have explored whether localized tissue impedance is associated with self-reports of pain during free living in older adults with knee OA. This provides the motivation for this effort. The current study aimed to determine if localized knee-tissue bioimpedance data collected from older adults with and without knee OA is a predictor of knee pain during free living. The following sections outline the wearable BIS system to collect free-living data, the selection and training of study participants, methods to collect self-report data during 7 days of free-living, and the statistical methods to evaluate bioimpedance data as a predictor of knee pain.

## II. Methods

### A. Wearable Knee Bioimpedance Sensing System

Localized knee-tissue bioimpedance data was collected from study participants using an electronic sensing system integrated into a commercially available textile knee brace (Swede-O Thermal Vent Open Knee Wrap Stabilizer). This sensing platform and wearable device have been validated during bench-top laboratory testing [26] and during free-living use by healthy adults [27]. The complete technical details of the wearable sensing system are available for interested readers [27], with a short technical summary presented here. Multi-frequency (8 kHz to 128 kHz) bioimpedance measurements were collected from two sites of the knee by the sensing platform. Measurements were captured with a MAX30001 integrated circuit (IC) [26] designed to drive/sense two tetrapolar configurations of Ag/AgCl electrodes interfaced to the tissues. The two sets of tetrapolar electrodes on the knee of a participant are shown in Fig. 1(b). The labels $E_{L}$ and $E_{T}$ in Fig. 1(b) correspond to the longitudinal and transverse configurations, respectively. These locations are hypothesized to capture fluid shifts in the knee (i.e. swelling) and OA associated structural changes (i.e. worn cartilage, bone spurs, and reduced joint spacing). The electrode locations in relation to the knee and corresponding OA symptoms are highlighted in Fig. 1(a). These changes are all expected to contribute to knee pain during free-living and motivate their selection for the sensing system.

The MAX30001 was configured to collect resistance and reactance at 5 discrete frequencies (8 kHz, 18 kHz, 40 kHz, 80 kHz, and 128 kHz) with a 8 $\mu$A current stimulus. A single set of bioimpedance measurements was collected every 2.5 minutes for the entire period that the device was powered and operating. The collected resistance and reactance measurements at each of the 5 frequencies were an average of 8 measurements captured sequentially at 64 Hz by the system and were stored on a micro-SD for later download and processing. The system electronics were populated on a custom printed circuit board (PCB) and this unit (with rechargeable battery) was integrated into an external pocket on the textile brace. This PCB and its external location in the brace when worn by a user is shown in Fig. 1(c).

### B. Study Participants

Older adults (average age = $73.5\pm 8.26$ years old) with and without knee OA ($N=20$, 10 with knee OA, 10 without) were recruited to participate in a 7-day data collection period for this study. This research and its activities were approved by The University of Alabama's Institutional Review Board (UA-IRB-18-013-ME). Exclusion criteria included (1) significant cognitive impairment, (2) knee replacement in knee with OA, (3) life-threatening illness, (4) diagnosed rheumatoid arthritis, fibromyalgia, of other rheumatologic disease, (5) speech or language problems that prevent interviews in English, and (6) individuals with any type of implantable medical device. For participants with OA, confirmation of disease was provided by their primary care physician or rheumatologist. A summary of the demographic characteristics of the study participants is provided in Table I detailing the average age, sex, brace size, and duration of time living with OA (where applicable). The age between groups was not significantly different ($p>0.05$). The study population was 85% women, with 40% and 45% women in the control and OA group, respectively. The average brace size worn by each group is also shown in Table I. For this comparison, small/medium (SM), large (L), two-extra large (2XL), three-extra large (3XL), four-extra large (4XL), and five-extra large (5XL) braces were given values of $1-7$, respectively. The overall average brace size worn within the sample was a 2XL, with the averages within control and OA groups being a 2XL and a 3XL. Median brace size for control (3) and OA (4) groups was not statistically significantly different as assessed by a Mann-Whitney U test.

**TABLE I:** Sample Characteristics

**Age**, range $57-89$ years		$73.5\pm 8.26$
Control		$71.4\pm 8.18$
OA		$75.6\pm 8.21$
**Sex**		
Control		
Male, no. ($\%$)		2 (10)
Female, no. ($\%$)		8 (40)
OA		
Male, no. ($\%$)		1 (5)
Female, no. ($\%$)		9 (45)
**Brace Size**, range SM (1) to 5XL (7)		$3.3\pm 1.81$
Control		$2.7\pm 1.57$
OA		$3.9\pm 1.91$
**Knee Circumference**, range 33.0 - 50.8 cm		$41.2\pm 4.4$
Control		$39.9\pm 3.8$
OA		$42.4\pm 4.7$

After screening for exclusion criteria, all participants completed an in-person interview to collect demographic, physical function, and health data. Participants with knee OA additionally provided information related to their arthritis symptoms. A summary of this data is shown in Table II for the 10 OA group participants. Four participants have been living with knee OA for $>10$ years, four for $5-10$ years, and two for $\leq 2$ years. Regarding mobility, 60% participants with knee OA reported having swelling within the last week, with 30% reported moderate to severe pain within the last month.

**TABLE II:** Summary of OA History, Weekly Symptoms, and Monthly Pain Self-Reported by Participants With Knee OA

	$1-2$ years	$3-5$ years	$6-10$ years	$11-15$ years	$15+$ years
How long have you had problems with your knee?	2	0	2	3	3
During the last week:					
	No Days	Few Days	Some Days	Most Days	All Days
Did you have swelling in your knee?	4	3	2	1	0
Can you straighten your knee fully?	2	0	0	0	8
Can you bend your knee fully?	2	0	1	0	7
During the past month:					
	None	Mild	Moderate	Severe	Extreme
How would you describe the arthritis pain you usually had?	1	6	1	2	0
	No Days	Few Days	Some Days	Most Days	All Days
How often did you have severe pain from your arthritis?	5	4	0	1	0

### C. Participant Training

Within a week of completing the eligibility screening and health questionnaire participants completed a 1 h training session with study personnel. This training provided a study overview, technology review, and detailed instructions on the setup and use of the wearable system. During training, participants were provided with a kit of materials, the wearable sensing system, and a user manual. The user manual included concise text descriptions with visual descriptions to guide participants through brace setup. Participants were shown how to correctly operate the system which required: connecting the battery to the system, cleaning the skin of their knee, placement of Ag/AgCl electrodes in the wearable, placement/tightening of the wearable on the knee, and recharging the battery after taking off the wearable.

Participants first observed these steps performed by the study personnel with specific reference to the user manual at each stage to highlight where participants could find this information if needed later. Next, participants completed each step with an opportunity to ask for further clarification. This training was not stopped until the participant successfully demonstrated each necessary step without direction or correction by study personnel. Training required approximately 1 h for each participant. The day after training, participants started the 7 days of data collection. After the 7 days, the kits were retrieved from the participant by the study personnel. The complete set of data collected over each day of use was downloaded for decoding and post-processing in MATLAB.

### D. Participant Pain Experiences

Each participant's individual pain experience during the 7 days of the study was captured using experience sampling methods (ESM). These are diary-like research procedures in which participants provide self-reports in real-time at random time points throughout a day of interest [28]. This methodology affords accurate assessment of participant experiences in their natural environment [29] and has been used in previous studies to investigate OA and its affects [30], [31]. In this work, the ESM protocol included 4 assessments per day when wearing the knee sensing system (capturing up to 28 prompts per participant over 7 days). The timing of calls was randomized within 3-hour blocks from 8 am to 8 pm. Each prompt required approximately 5 minutes and captured where the participant was currently located, what activities they had engaged in since the last call (or waking up), their experience with the brace, and their momentary pain experience. For reporting of their pain experience the participants were asked if they had experienced pain since they woke or since the last phone call (“yes or no”) and their level of pain at that moment on a scale from 0 (no pain) to 4 (extreme pain). If pain was reported the location of pain was also recorded. Over the entire study period, the response rate to the ESM phone calls from the 20 participants was 94.2%.

### E. Data Post-Processing

#### 1) Bioimpedance Artifact Cleaning

Once the wearable system and its logged data were obtained from the participants, post-processing included assessing the data quality, removing data artifacts, and reducing data dimensionality. Bioimpedance data was processed by a binary decision tree algorithm to classify it as artifact-free or as a data artifact. Data collected during unsupervised free living can have errors introduced by motion artifacts, electrode disconnect events, electrode aging, cabling damage/disconnects, and electronics/sensor damage. These artifacts need to be removed prior to analysis because they do not represent the tissue impedance of the participant being measured. A threshold method for artifact identification was presented by Freeborn and Critcher [32] which has been modified in this work to include additional steps to check for proper wearable use, check for sensor malfunction events, and remove ESM periods that do not have sufficient data to generate an impedance metric. The summarized binary decision tree steps are below:

• Do the number of distinguishable battery discharge events align with the participant's reported number of days wearing the system?
• Is the on-board impedance test model measurements within $\pm 10\%$ of expected values?
• Is the phase angle ($\Theta$) within the range: $-50^\circ$ $< $ $\Theta$ $< $ $0^\circ$?
• Is the resistance ($R$) within the range: 0 $\Omega$ $< $ $R$ $< $ 212 $\Omega$?
• Is amount of artifact free data within the call period $>$ 25%?
• Do at least 3 of the 4 call periods within that day have $>$ 25% artifact free data?

**Fig. 2.**
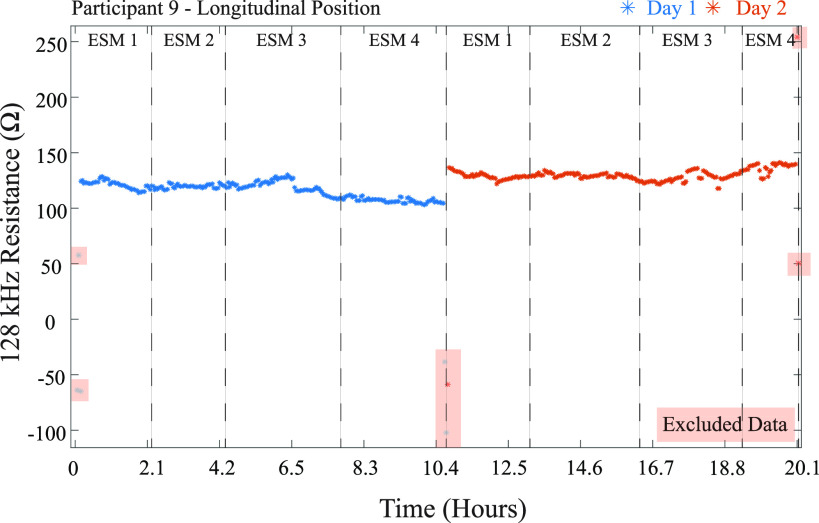
Participant 9 longitudinal resistance for days 1 and 2 of the study, including both artifact-free and artifact classified data.

If the answer to any step was “no” the data was classified as an artifact and removed from further processing. Further details about this process are provided in [27], [32] for interested readers. To illustrate this process, Fig. 2 outlines its application to a subset of data from a single study participant. Fig. 2 presents the 128 kHz resistance captured by the sensing system when worn for approximately 20 hours across 2 consecutive days. Day 1 and 2 datasets are represented as blue and red datum, respectively. Notice that there are 5 sets of data in this figure highlighted by red boxes. This is data classified as artifacts due to exceeding the threshold values and will be removed from further processing and analysis steps.

To compensate for differences in measured tissue segments between participants associated with variations in knee size and brace sizing, impedance measurements were transformed into per-length values such that:

\begin{align*}
\mathrm{PLR_{i}} & = \frac{R_{i}}{L} \tag{1}
\\
\mathrm{PLX_{i}} & = \frac{X_{i}}{L} \tag{2}
\end{align*}

where $\mathrm{PLR_{i}}$ and $\mathrm{PLX_{i}}$ represent the per-length resistance and reactance, respectively, at frequency $i$ with units $\frac{\Omega }{meter}$ and $L$ is the distance between voltage sensing electrodes in the brace for both the longitudinal and transverse electrode locations. A similar approach has been applied in studies of body composition using bioimpedance data, scaling bioimpedance metrics by participant height, weight, BMI, or their combination [33], [34].

#### 2) Bioimpedance Dimensionality Reduction

The PLR and PLX data at each frequency were reduced to a single mean and coefficient of variation (CV) for each ESM period to reduce the dimensionality of the collected data. This is to provide one metric for the central tendency and one metric for the variation to represent each ESM period for the pain prediction models. After this reduction, the tissue bioimpedance across 7 days was represented by up to 28 datapoints each for the mean and CV.

#### 3) Pain Experiences

The participant self-reports of their pain experiences captured with the ESM protocol were used to generate a binary “pain” or “no pain” value for each call period. The self-reported pain experiences captured during the ESM protocol are summarized in Table III. Note, participants who did not report any pain over the 7 days are not listed. In this table empty cells represent days where no pain experiences were reported. The numbers within cell parentheses represent the reported knee pain (on the scale of $0-4$) for that call period ($1-4$). For example, a cell with (3,2,2,0) captures that a participant reported pain at a scale of 3 during their first call, 2 during the second, 2 during the third, and 0 at their final call. If a cell contains an “X,” this captures that the participant reported knee pain between ESM calls but was not currently experiencing pain. An asterisk denotes pain was reported by the participant but no corresponding bioimpedance data was available (due to the artifact cleaning process).

**TABLE III:** Pain Summary of Study Participants From 7-Days of ESM Reporting

		Day						
Part.	Group	1	2	3	4	5	6	7
1	OA		(0,0,0,1)					
3	Control					(2,0,0,0)		
4	OA				(0,0,0,1)			
9	OA		X	X	X		X	X
10	OA			X*			(1,0,0,0)*	
11	OA		(2,0,0,0)					X
15	OA	(0,3,0,3)	(0,1,0,0)	(3,2,2,0)	(0,1,2,1)		(3,0,2,0)	(1,1,2,0)
16	OA	(1,1,0,0)	(2,0,2,3)	(3,2,0,1)	(0,1,1,2)	(0,3,2,3)	(0,0,1,1)*	(0,2,2,3)
17	OA	(1,0,0,0)				X		
18	OA	X*					X*	

$^*$Pain data reported, but no corresponding impedance data

## III. Results

The reduced dimensionality impedance data for all study participants are provided in Tables I and II in the Supplemental Material, for both the longitudinal and transverse electrode locations. For each of the 20 participants, there are four impedance metrics: $PLR_{\text{128}\;\text{kHz}}$ average, $PLR_{\text{128}\;\text{kHz}}$ CV, $PLX_{\text{40}\;\text{kHz}}$ average, and $PLX_{\text{40}\;\text{kHz}}$ CV. Each column corresponds to a call-period of reduced data per participant. For the longitudinal dataset there were 418 datapoints for each metric, with an average of 20.9 datapoints per person. For the transverse location there was a total of 409 datapoints with an average of 20.45 datapoints per person. Each participant's number of datapoints varies according to the number of days the wearable was worn, the number of successful ESM assessments, and the amount of data after the artifact cleaning process.

### A. Multi-Level Modeling

To determine if bioimpedance data representing localized knee tissue characteristics can predict participants knee pain a multi-level modeling (MLM) approach was adopted. Multi-level modeling is commonly used for hierarchical and longitudinal data structures. In the case of the dataset in this work, there are individual datapoints (e.g. impedance metric of each ESM period) nested within participants and each participant belongs to either the control or OA group (Control $=0$ and OA $=1$). Multi-level modeling allows for the investigation of within person variance and between person variance in the collected bioimpedance data and how it pertains to their pain experiences. A generalized mixed model analysis is used because the outcome variable, presence of pain, was a dichotomous variable.

#### 1) Correlated Bioimpedance Metrics

**Fig. 3.**
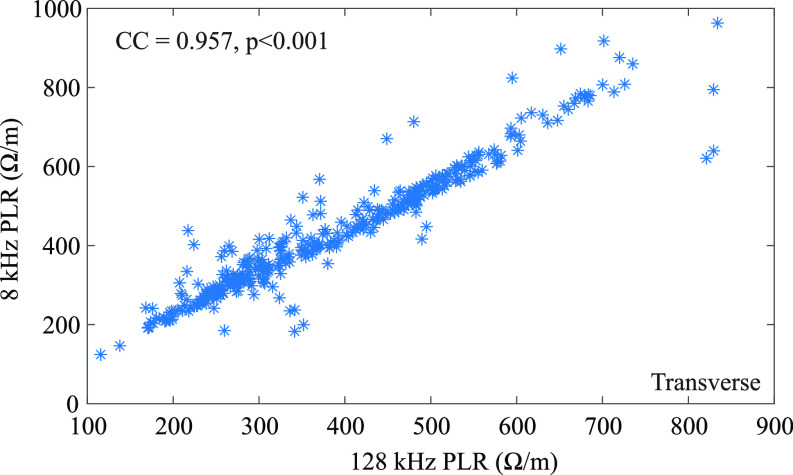
Transverse 128 kHz and 8 kHz scaled resistance data plotted to illustrate correlation.

To identify bioimpedance metrics to use in the MLM, a Spearman's rank correlation was applied to the resistance and reactance means and CV at 8 kHz, 40 kHz, and 128 kHz for longitudinal and transverse data independently. Statistically significant (p $< 0.05$) correlations (CC $>\pm 0.5$) were identified across all mean resistance and reactance metrics for both electrode configurations. The largest correlation (CC $>0.9$) was reported for the mean resistance values. To visualize, the correlation (CC = 0.957, $p< 0.001$) between mean 8 kHz and 128 kHz resistance metrics is shown in Fig. 3 for the transverse electrode location. As a result of the high correlation between resistance metrics, only a single frequency (128 kHz) was included in the MLM analysis. Additionally, to ensure the correlations between the resistance and reactance metrics (CC $>\pm 0.4$) did not effect the MLM results, these datasets were also analyzed independently.

#### 2) Model Analysis

The associations among bioimpedance metrics and pain (i.e. in between calls or at the same phone call) were examined through various multilevel models that nested the 28 datapoints (level 1 - ESM calls and reduced bioimpedance metrics) within participants (level 2). For both longitudinal and transverse datasets, a null model was initially run to determine the appropriateness of applying multi-level analysis. This null model estimates the within- and between-person variance in the data. The intraclass correlation (ICC) was 0.586 and 0.594 for the longitudinal and transverse electrode location datasets, respectively. This ICC indicates 41.4% and 40.6% of the variance occurred within individuals. This supports that the multi-level analysis approach is appropriate based on the sufficient within-persons variances.

Next, the MLM analysis treated pain as the outcome variable with bioimpedance metrics and grouping variable as the predictors. This model, referred to as model 1, is given by:

\begin{equation*}
P_{ij} = \gamma _{00}+\gamma _{01}\text{Group}_{j}+\gamma _{10}\text{PLZ}_{ij}+\gamma _{20}\text{CV}_{ij}+\mu _{0j} \tag{3}
\end{equation*}

where $P_{ij}$ is the predicted log-odds of pain (e.g. probability of currently experiencing knee pain), $\gamma _{00}$ is the participant-level grand mean, Group (represented as a binary value with $OA=1$ and $Control=0$) is included as a predictor, $PLZ_{ij}$ is the per-length impedance metric (either resistance or reactance) included as a predictor, $CV_{ij}$ is the coefficient of variation (corresponding to the PLZ metric) included as a predictor, and $\mu _{0j}$ is the grouping variation from the grand mean value. The subscript $i$ corresponds to the individual data point within a participant's data set and the subscript $j$ corresponds to the participant.

Note that the resistance and reactance metrics were run independently of each other for both the longitudinal and transverse electrode locations, resulting in a total of 8 different models analyzed. From this analysis, only the grouping variable (e.g. having knee OA or not) was a predictor for pain. These results are summarized in Table IV. For the longitudinal electrode position, participants within the OA group were $42-54$ times more likely to be in pain as compared to the healthy/control group. For the transverse electrode location participants within the OA group were $33-36$ times more likely to be in pain.

**TABLE IV:** Associations Among Pain as a Function of Longitudinal and Transverse Bioimpedance Metrics and Group

Longitudinal Electrode Position									
	Model 1			Model 2			Model 3		
	$\beta$ (Exp.)	SE	p	$\beta$ (Exp.)	SE	p	$\beta$ (Exp.)	SE	p
Pain									
$R_{\mathrm{128_{kHZ}}}$ $\mu$	0.004(1.004)	0.0023	0.127	$-0.002(0.998)$	0.0017	0.273	0.005(1.005)	0.0027	0.079
$R_{\mathrm{128_{kHZ}}}$ cv	$-0.004(0.996)$	0.0511	0.934	$-0.185(0.831)$	0.0959	0.054	0.003(1.003)	0.0547	0.963
Group (OA)	3.996(54.37)	1.364	0.004*	$-1.267(0.282)$	2.513	0.614	-	-	-
Group × $R_{\mathrm{128_{kHZ}}}$ $\mu$	-	-	-	0.007(1.007)	0.0033	0.038*	-	-	-
Group × $R_{\mathrm{128_{kHZ}}}$ cv	-	-	-	0.188(1.206)	0.1102	0.09	-	-	-
Pain									
$X_{\mathrm{40_{kHZ}}}$ $\mu$	$-0.007(0.994)$	0.012	0.578	0.019(1.019)	0.0084	0.002	$-0.013(0.987)$	0.0129	0.305
$X_{\mathrm{40_{kHZ}}}$ cv	0.005(1.005)	0.023	0.818	$-0.035(0.965)$	0.0241	$0 .145$	0.008(1.008)	0.0240	0.737
Group (OA)	3.75(42.533)	1.232	0.002*	1.872(6.501)	1.4677	0.203	-	-	-
Group × $X_{\mathrm{40_{kHZ}}}$ $\mu$	-	-	-	$-0.031(0.969)$	0.0155	0.044*	-	-	-
Group × $X_{\mathrm{40_{kHZ}}}$ cv	-	-	-	0.043(1.044)	0.0336	0.201	-	-	-
Transverse Electrode Position									
	Model 1			Model 2			Model 3		
	$\beta$ (Exp.)	SE	p	$\beta$ (Exp.)	SE	p	$\beta$ (Exp.)	SE	p
Pain									
$R_{\mathrm{128_{kHZ}}}$ $\mu$	0.004(1.004)	0.0028	0.121	$-0.028(0.972)$	0.0143	0.052	0.007(1.007)	0.0028	$0.014*$
$R_{\mathrm{128_{kHZ}}}$ cv	$-0.028(0.972)$	0.0766	0.714	$-0.569(0.566)$	0.1742	0.001	$-.004(0.996)$	0.0766	0.959
Group (OA)	3.507(33.343)	1.228	0.005*	$-9.228(9.825E-5)$	5.000	0.066	-	-	-
Group × $R_{\mathrm{128_{kHZ}}}$ $\mu$	-	-	-	0.035(1.035)	0.0146	0.018*	-	-	-
Group × $R_{\mathrm{128_{kHZ}}}$ cv	-	-	-	0.566(1.761)	0.1904	0.003*	-	-	-
Pain									
$X_{\mathrm{40_{kHZ}}}$ $\mu$	$-0.017(0.983)$	0.0095	0.067	0.307(1.359)	0.0682	$< 0.001$	$-0.023(0.997)$	0.011	0.037*
$X_{\mathrm{40_{kHZ}}}$ cv	$-0.001(0.999)$	0.0114	0.928	0.004(1.004)	0.0030	0.200	$-0.002(0.998)$	0.0142	0.886
Group (OA)	3.573(35.616)	1.194	0.003*	$-1.543(0.214)$	1.9307	0.425	-	-	-
Group × $X_{\mathrm{40_{kHZ}}}$ $\mu$	-	-	-	$-0.330(0.719)$	0.0691	$< 0.001$*	-	-	-
Group × $X_{\mathrm{40_{kHZ}}}$ cv	-	-	-	$-0.006(0.994)$	0.0146	0.694	-	-	-

Because the majority of pain reports were within the OA group, the interaction between the per length impedance metrics and group was assessed using an additional model to explore between groups differences. This expanded on (3) by adding an interaction term to assess moderating effects of grouping on the impedance metric-pain association. This model, referred to as model 2, is given by:

\begin{align*}
P_{ij} = & \gamma _{00}+\gamma _{01}\text{Group}_{j}+\gamma _{10}\text{PLZ}_{ij}+\gamma _{20}\text{CV}+\mu _{0j} \\
& + \gamma _{11}\text{PLZ}_{ij}*\text{Group}_{j}+\gamma _{21}\text{CV}_{ij}*\text{Group}_{j} 
\tag{4}
\end{align*}

**Fig. 4.**
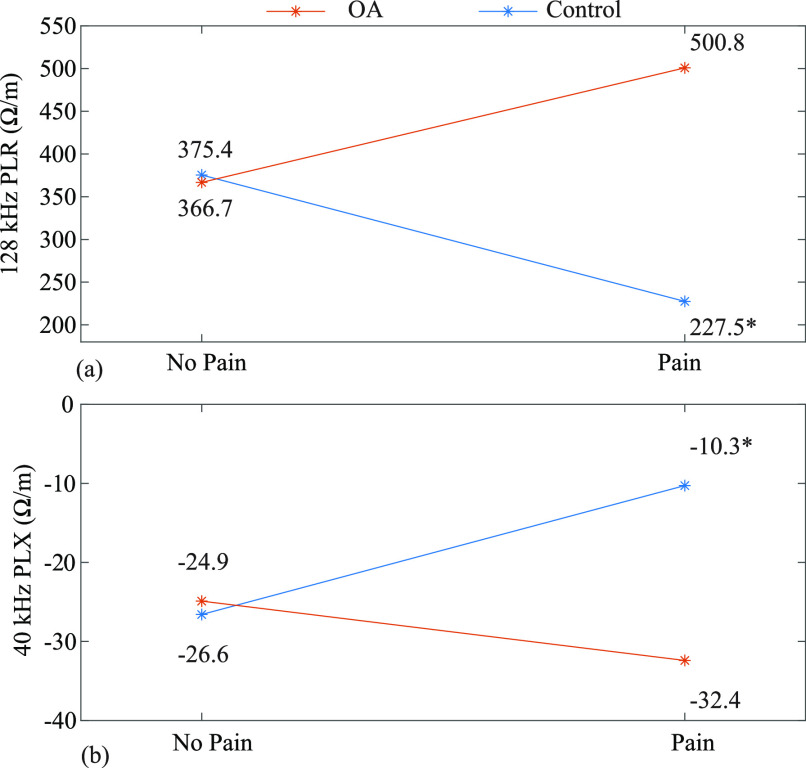
(a) Moderating effects of group in the association of knee pain with transverse per-length resistance at 128 kHz (b) Moderating effects of group in the association of knee pain with transverse per-length reactance at 40 kHz.

The model 2 results, summarized in Table IV, indicate the transverse electrode location shows significant interaction terms for PLR mean x group ($p=0.018$), PLR CV x group ($p=0.003$), and PLX x group ($p< 0.001$). The significance of the PLR mean and grouping term indicate that an increase in per-length resistance within the OA group, significantly increases the probability of being in pain by 1.035 times. To visualize this significant interaction term, the group means for both pain and no pain experiences are shown in Fig. 4(a). This Fig. reports on average the PLR within the OA group was higher during self-reports of pain than during periods of no pain. Because this interaction is significant, it suggests an increase in PLR corresponds to a higher probability of being in pain for the OA group. The significance of the PLX and grouping interaction term corresponds to a decrease in PLX at 40 kHz within the OA group, significantly increases the probability of being in pain by 0.719 times, with this interaction shown in Fig. 4(b).

Further, the model 2 for the longitudinal electrode configuration indicate that PLR and group interaction were significant ($p=0.038$). That is, an increase in PLR at 128 kHz within the OA group, significantly increases the probability of being in pain by 1.007 times. The PLX and group interaction of this electrode position was also significant ($p=0.044$), indicating that a decrease in per-length reactance at 40 kHz (within the OA group) significantly increases the probability of being in pain by 0.969 times.

#### 3) Control Group Reported Pain

An unexpected result of the model 2 analysis interaction plots, shown in Fig. 4(a) and (b), was the pain mean value for the control group dataset (designated with a ‘*’). It was expected that only participants within the OA group would report pain events, but there was a single report of pain by a control group participant (participant 3). This single pain report is hypothesized to be a result of irritation from the brace and not localized knee pain due to OA (since this participant does not have knee OA). Based on Fig. 4(a) and (b) this single datapoint leads to a reporting of a trend opposite of the OA group average for a pain report for both the PLR and PLX metrics.

It is hypothesized this single metric could have an undesired effect on the MLM results since the aim of this study was to investigate bioimpedance as a predictor for OA-related pain, not other pain associations. To eliminate the effects of this single datapoint and to investigate its effect on model 2 results, a third MLM model was run. Model 3 analyzed the mean and CV impedance metrics as a predictor for the probability of active pain. This is the same as model 2 but only using the OA group data for analysis. This choice eliminates the effect of the control group pain variable reported by participant 3 that was suspected to be unrelated to OA knee-pain. It was hypothesized, that if statistical significance remained across model 2 and 3 then this single metric did not have an effect on overall analysis.

The results for model 3 analysis are summarized in Table IV. Notice, there are no interaction term results shown for model 3, as a result of only using the OA group data. For the longitudinal data, neither mean PLR or PLX parameters were significant, when model 2 results did show significance for the interaction of group and these metrics. This differences in statistical significant between models 2 and 3 suggest that the single datapoint from the control group is the source of the difference. The transverse electrode location results align with model 2 results, with the exception of the PLR CV interaction term. Again, this is a result of including the control group pain datapoint in the analysis.

The significance in mean PLR and PLX metrics as predictors of participant pain from model 2 are confirmed by the results of model 3. Fig. 4(a) and (b) summarize the group means of pain and no pain experiences within the OA group for the PLR and PLX metrics. The PLR and PLX group averages were 366.7 $\Omega /\text{m}$, 500.8 $\Omega /\text{m}$, $-24.9$ $\Omega /\text{m}$, and $-32.4$ $\Omega /\text{m}$, for the no pain and pain groups respectively.

## IV. Discussion

**Fig. 5.**
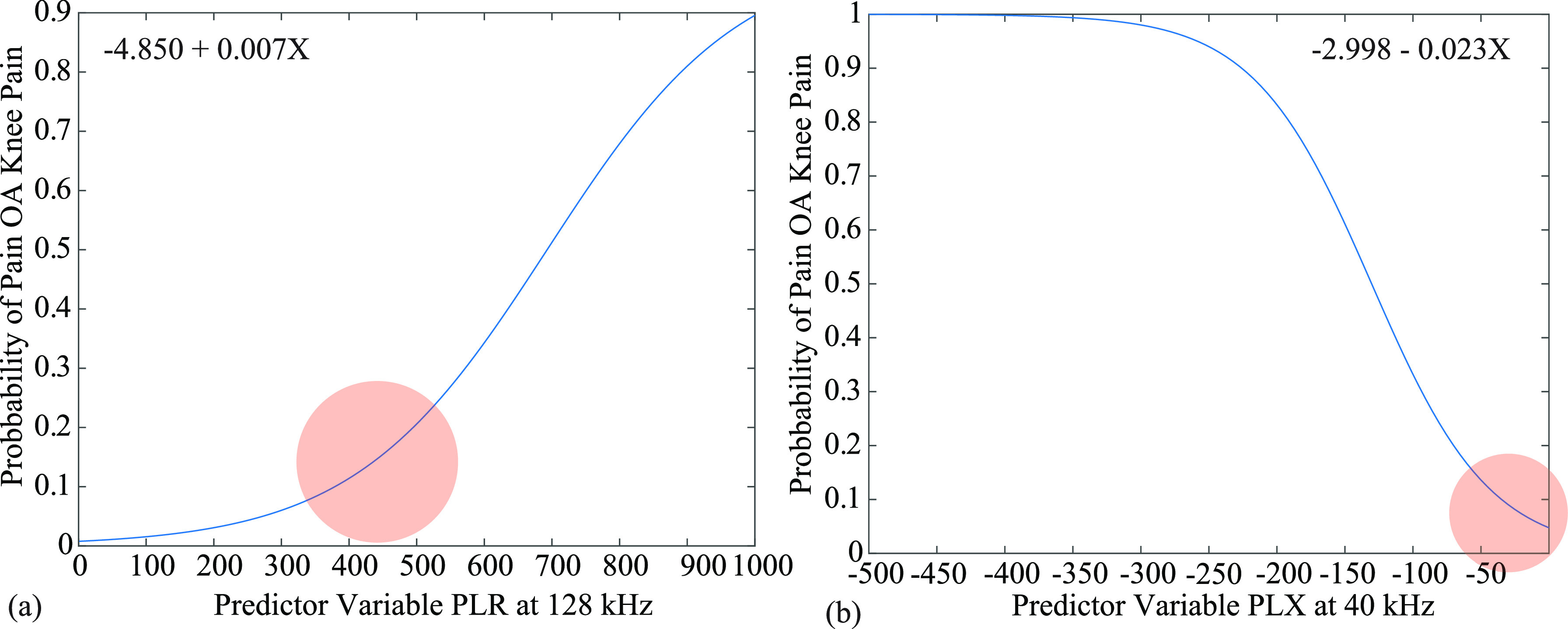
(a) Conditional probability for the transverse PLR (128 kHz) and (b) PLX (40 kHz) metrics as predictors for OA knee pain.

The results from this study suggest bioimpedance metrics can be used as a predictor for active pain experiences for those who suffer from knee OA. The results from model 3 confirmed the average PLR at 128 kHz and PLX at 40 kHz for the transverse electrode position were statistically significant predictors of the probability of active knee pain. Using the intercepts and coefficients produced from the SPSS output, the probability of active OA-knee pain for the transverse electrode position is shown in Fig. 5. The intercepts and coefficients for the PLR and PLX parameters were $\beta _{0}=-4.850$, $\beta _{1}=0.007$, and $\beta _{0}=-2.998$, $\beta _{0}=-0.023$, respectively. Based on these plots, the model suggests that increases in PLR at 128 kHz and decreases in PLX (increased magnitude) at 40 kHz increase the probability of OA-knee pain. The red circle on each plot highlights the PLZ parameter ranges in this study.

**Fig. 6.**
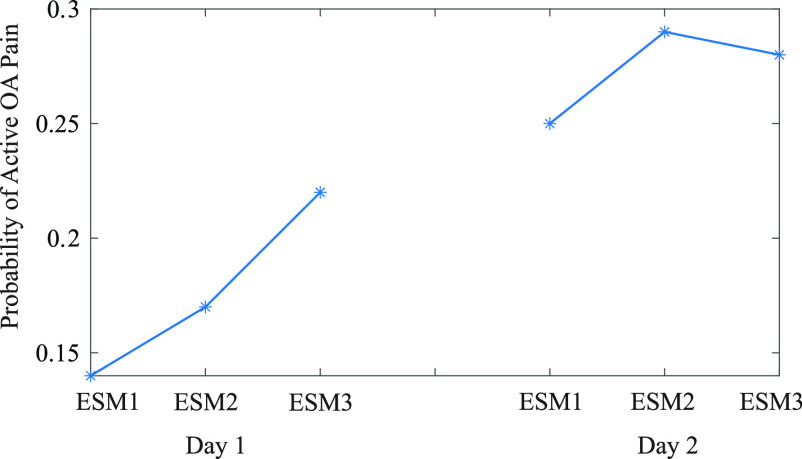
Probability of active OA pain for participant 15 d 1 and 2 data.

An example application of this model is shown in Fig. 6 where the probability of active OA pain is shown for days 1 and 2 of the study using the 128 kHz PLR metric. For day one, the participant's PLR increases, increasing their probability of active OA pain from 14% to 22%. For day 2 the participant had the highest probability of pain in the middle of the day at 29% compared to the 25% and 28% during the times before and after. This ability to track the increases and decreases in probability of active OA knee pain based on the measured PLZ metrics could provide physicians and treatment providers a reliable, non-invasive, and unobtrusive approach to track and monitor short-term changes (spanning minutes, hours, and days) related to OA and the pain experiences by the patients, allowing for a data-driven personalized treatment approach.

A notable outcome is only a single resistance metric was required to predict the probability of OA knee pain. With very high correlations ($CC>0.9$) between tissue resistances of the frequencies (8 kHz and 128 kHz), future studies related to pain prediction may focus on collecting only a single-frequency resistance (which has implications to reduce the hardware required for a wearable system). The MLM results also highlighted that only the transverse electrode location metrics were significant predictors for OA-related knee pain, not the longitudinal electrode position metrics. The transverse metrics reported an increase in PLR at 128 kHz associated with an increased probability of OA pain. This suggests that only impedance measurements localized to the knee joint, as compared to the longitudinal electrode position that also included skeletal muscle, are needed in the monitoring of probability of OA-related knee pain.

Neves et al. collected transverse knee measurements on 32 males with and without OA and reported OA groups having resistance values of approximately 850 $\Omega$ and 525 $\Omega$ [18]. Further reporting that increased resistance correlated to OA disease progression (as assessed via the Dejour scale) [18]. While Neves et al. did not specifically investigate OA related pain, additional studies in the literature have reported that participants with frequent pain experiences were more likely to have a higher severity of knee OA (as classified by Kellgren Lawrence grades) [35]. Limitations of the results presented by Neves et al. include the use of a bipolar electrode configuration. In a bipolar setup, the measurements reflect both the tissue impedance and the tissue/electrode interface impedance (not just the tissue). The measurements collected in this work utilized a tetrapolar electrode configuration, which reduced the effects of the electrode/skin interface impedance. As a result of using this electrode configuration, changes in impedance measurements reported in this work are expected to be more strongly associated with the physiological changes and not electrode/skin interface impedance.

Many studies in the literature, while not specifically investigating pain, have investigated differences between injured and healthy knees. Reports from these studies note reduced resistance is associated with increased edema (swelling) [19], [21], [24], [25], [36]. The increase in PLR at 128 kHz associated with an increased probability of OA pain reported in this work does not align with these reports in the literature. While this disparity could be a result of the different electrode positions used (transverse vs longitudinal), it highlights the need to further interrogate the common association of increased swelling to a decrease of resistance and how geometry of the measurement site and electrode configuration influences measurements. This work did not aim to elucidate the mechanism of physiological change associated with OA and its relationship to the reported bioimpedance measurements, but supports the need for further research to investigate these associations.

It is important to note that the participants recruited for our study introduced a limitation to the pain prediction. From a review of the overall pain reports summarized in Table III, not all participants with physician diagnosed knee-OA experienced knee pain within the past week or month. So while members of this group did experience chronic knee pain as a result of their OA, chronic pain suffers can have periods or “flares” that denote periods of active intense pain differentiated from constant dull pain [37], [38]. It is hypothesized, that the differences in the pain events reported within the OA group could be a result of these “flares”. Based on the self-reports of pain severity within the past month, noted in Table II, only 30% of the OA group experienced moderate to severe pain. This suggests that the majority of participants were in a latent pain phase, between an active or “flare” period of extreme pain. The disproportionate amount of pain data as compared to the number of participants within the OA group could have an impact on the results presented in this work. While the model in this work notes that larger transverse resistance increases probability of knee-pain, this effect could be even more significant in populations with greater pain. While these preliminary results suggest bioimpedance as a potential predictor for knee OA-related pain, limitations of this study include both the small number of participants and limited pain reports across the OA group. To test this hypothesis future studies are needed that collect knee tissue bioimpedance from a greater number of participants with greater active pain.

Another limitation of this study is the use of only a single data type (bioimpedance). The bioimpedance data was collected in a free-living environment where movement profiles could affect the resulting impedance trends. Studies have shown various types of activities, including walking and bicep curls, can result in changes in impedance measurements ranging from $6-8$ $\Omega$ for measurements collected on the knee [39] and bicep [40]. Postural changes can also cause fluid shifts in the body that can result in gradual drift in impedance measurements over time [41]. While the post-processing described in Section II-E1 aimed to identify artifacts from electrode/cable disconnect events that could impact analysis/interpretation there was no processing to identify artifacts that may be a result of contraction or body position. Future analysis should aim to eliminate movement/postural artifacts in the bioimpedance data by incorporating other data modalities (e.g. acceleration, rotational data) into the analysis.

## V. Conclusion

This work collected free-living bioimpedance data using a wearable knee sensing system from populations of older adults with and without knee OA to evaluate if localized tissue bioimpedance was a potential predictor of knee pain. Using MLM to generate a predictor model, it is observed that an increase in per-length resistance and a decrease in per-length reactance of transverse tissue impedance significantly increased the probability of being in pain by 1.007 and 0.969 times for people with OA. There was approximately a 20 $\Omega$ increase in resistance and a 7.5 $\Omega$ decrease in reactance for pain reports within the OA group. These results support the future investigation of bioimpedance marker of knee pain for those with knee OA.

## Supplemental Material

The complete set of bioimpedance data that was utilized within the statistical models of this paper are provided in the Supplementary Materials in Tables I and II for the longitudinal and transverse measurement locations, respectively.

Supplementary materials
