# Supplementary material for: Localized Multi-Site Knee Bioimpedance as a Predictor for Knee Osteoarthritis Associated Pain Within Older Adults During Free-Living
Source: IEEE Open J Eng Med Biol. 2023 Mar 13;4:1–10. doi: 10.1109/OJEMB.2023.3256181 (PMC10151013; doi:10.1109/OJEMB.2023.3256181)
Supplement: Supplementary materials [file supp1-3256181.pdf]

Supplementary Materials

Localized Multi-Site Knee Bioimpedance as a Predictor for Knee Osteoarthritis Associated Pain within Older Adults During Free-Living

Shelby Critcher, *Student Member, IEEE,* Patricia Parmelee, and Todd J. Freeborn*, Jr., *Senior Member, IEEE*

THIS Supplementary Material provides the complete set of bioimpedance data that was utilized within the statistical models of this paper are provided in Tables I and II for the longitudinal and transverse measurement locations, respectively. For both measurement locations there are four impedance metrics: PLR_{128kHz} average, PLR_{128kHz} CV, PLX_{40kHz} average, and PLX_{40kHz} CV. Each column in the tables corresponds to the specific ESM call period. With 4 ESM periods per day for the 7 days there is a maximum of 28 ESM periods for which bioimpedance data was captured. The bioimpedance metrics within each column represents the impedance metric using only data from within that ESM period. Cells that are blank represent ESM periods that do not have a bioimpedance metric associated with them. For the longitudinal and transverse electrode positions there was 74.6% and 73% of total 560 (28 datapoints $\times$ 20 participants) datapoints expected across all participants.

Longitudinal Electrode																													
Participant	Metric	Datapoint																											
		1	2	3	4	5	6	7	8	9	10	11	12	13	14	15	16	17	18	19	20	21	22	23	24	25	26	27	28
1	PLR 128 kHz	479.00	486.20	293.28	454.36	495.13	512.98	516.36	520.66	504.54	529.06	532.91	521.95					482.61	492.47	450.37						514.31	498.97	476.40	
	PLR CV 128 kHz	3.54	12.44	10.16	16.32	3.10	2.37	2.08	1.88	3.33	2.87	3.62	4.19					2.77	7.77	23.06						1.69	6.58	9.32	
	PLX 40 kHz	−92.80	−93.24	−58.23	−87.86	−82.57	−81.98	−78.47	−72.79	−86.37	−88.58	−81.10	−81.80					−77.10	−80.10	−83.04						−81.86	−74.84	−75.86	
	PLX CV 40 kHz	−6.36	−16.96	−26.50	−15.91	−4.64	−4.83	−6.38	−4.32	−5.81	−5.32	−5.85	−6.43					−3.73	−7.48	−10.11						−4.82	−6.78	−10.88	
2	PLR 128 kHz	1015.21	945.87	949.56	1015.75	985.98	968.81	957.43	1029.57																				
	PLR CV 128 kHz	4.88	2.16	3.20	6.18	3.52	3.31	3.54	2.54																				
	PLX 40 kHz	−74.37	−65.78	−68.76	−83.77	−79.64	−79.22	−79.32	−89.30																				
	PLX CV 40 kHz	−5.81	−6.73	−9.01	−6.40	−6.97	−5.22	−7.53	−6.23																				
3	PLR 128 kHz	732.87	769.59	792.54	726.20	759.67	720.62	704.08	677.41	669.08	691.86	684.53	709.24	904.92	884.04	859.68	792.61	635.02	647.02	705.89	685.70	766.62	826.18	786.21	770.72	525.12	482.82	470.02	457.24
	PLR CV 128 kHz	2.88	4.23	4.13	2.22	3.87	2.70	2.44	2.53	2.80	2.65	2.65	3.64	2.94	2.24	2.91	3.72	3.30	4.74	3.51	3.36	8.37	3.61	2.77	3.11	5.37	4.41	5.01	4.65
	PLX 40 kHz	−75.35	−92.53	−92.01	−172.22	−72.81	−61.68	−66.14	−65.10	−64.98	−71.57	−76.63	−96.93	−105.58	−99.14	−109.49	−111.45	−71.74	−80.13	−85.81	−85.30	−97.64	−140.30	−134.47	−115.39	−49.39	−53.07	−47.85	−47.51
	PLX CV 40 kHz	−6.83	−7.27	−5.39	−23.74	−8.62	−5.27	−7.83	−5.44	−7.54	−7.10	−7.68	−12.69	−8.08	−5.93	−18.67	−6.86	−9.02	−8.30	−8.28	−7.33	−12.44	−11.02	−9.71	−7.95	−7.44	−5.67	−9.91	−10.19
4	PLR 128 kHz	968.74	1014.91	944.88	895.54	857.92	791.31	775.77	778.27	873.22	892.77	891.43	877.25	985.59	974.68	902.34	842.54	864.89	853.79	803.70	867.12	881.14	967.86	853.47	848.63	970.57	924.24	881.92	886.54
	PLR CV 128 kHz	1.80	2.32	5.03	3.28	2.83	1.69	1.76	2.31	2.98	3.23	2.81	2.51	0.86	3.08	3.54	1.98	0.43	3.66	2.65	4.12	2.43	3.09	4.96	6.52	4.82	4.31	2.80	3.71
	PLX 40 kHz	−54.88	−59.28	−54.99	−53.99	−45.39	−37.71	−37.50	−37.50	−50.27	−46.80	−47.31	−44.86	−58.92	−56.65	−47.18	−41.02	−52.49	−50.53	−46.75	−60.81	−49.58	−56.73	−47.36	−40.58	−56.81	−49.56	−48.90	−47.69
	PLX CV 40 kHz	−6.41	−5.61	−8.56	−7.42	−6.57	−3.31	−3.75	0.00	−31.73	−8.97	−7.39	−6.82	−4.81	−9.33	−9.99	−9.21	0.00	−9.73	−11.70	−12.43	−7.59	−6.61	−14.00	−10.60	−8.40	−7.43	−7.74	−7.62
5	PLR 128 kHz	1184.85	1136.79	1138.03	1161.71	1296.92	1206.56	1206.88	1199.65					1191.81	1064.44	1012.58	915.29	1007.31	956.42	962.68	911.22	971.02	1000.25	974.20	974.54	977.02	918.18	932.04	912.03
	PLR CV 128 kHz	1.53	1.97	2.37	2.22	1.26	5.70	1.90	1.95					1.92	12.21	11.27	33.70	3.71	3.78	2.60	4.32	2.50	1.67	1.88	2.81	3.01	2.52	2.14	2.58
	PLX 40 kHz	−85.58	−88.40	−92.49	−102.20	−112.07	−104.62	−111.43	−110.46					−87.16	−85.28	−97.29	−86.44	−89.61	−98.06	−100.41	−88.42	−93.95	−105.99	−103.28	−107.37	−75.45	−80.18	−82.74	−76.26
	PLX CV 40 kHz	−4.45	−6.40	−7.24	−5.88	−2.78	−9.58	−3.57	−3.45					−5.73	−12.63	−9.17	−18.51	−10.73	−6.38	−9.20	−10.09	−10.72	−6.57	−6.62	−9.96	−5.14	−11.01	−7.13	−7.61
6	PLR 128 kHz	905.26	872.62	906.84	1057.91	933.03																							
	PLR CV 128 kHz	2.31	2.95	5.67	7.59	7.46																							
	PLX 40 kHz	−87.58	−87.81	−138.68	−184.31	−97.49																							
	PLX CV 40 kHz	−4.03	−3.91	−20.50	−12.32	−14.55																							
7	PLR 128 kHz	496.84	470.25			457.68	424.80	414.61	398.66	493.82	462.33	459.40		530.30	503.02	487.92	506.72	513.12	522.60	519.37	506.45	499.21	491.12	477.69	466.15	508.58	491.92	479.77	
	PLR CV 128 kHz	3.24	3.44			5.60	5.65	4.31	10.49	2.44	8.23	5.14		2.27	2.51	3.97	4.40	5.47	5.95	2.45	3.16	2.67	4.47	2.47	2.05	6.10	5.10	2.76	
	PLX 40 kHz	−86.63	−87.18			−101.26	−91.29	−91.25	−90.90	−110.64	−110.68	−95.10		−108.57	−93.68	−96.66	−105.76	−139.98	−127.43	−113.33	−113.49	−101.34	−98.43	−92.22	−91.86	−107.57	−88.04	−81.69	
	PLX CV 40 kHz	−10.20	−7.49			−6.16	−7.20	−7.36	−15.36	−3.56	−6.63	−3.95		−3.16	−6.32	−9.03	−6.79	−11.16	−7.18	−5.97	−3.71	−5.30	−6.67	−2.70	−2.11	−7.82	−8.70	−5.81	
8	PLR 128 kHz	863.77	831.96	785.02		909.37	805.98	722.13	754.31	810.76	754.35	682.87	727.12	659.02	670.00	671.00	644.40	753.17	739.67			712.67	755.40	770.57		792.62	789.44	770.60	789.91
	PLR CV 128 kHz	1.74	2.60	5.08		3.88	5.88	2.96	2.18	3.09	5.03	8.41	3.54	4.56	4.73	2.33	4.68	2.57	3.05			5.30	3.65	3.61		4.11	4.61	3.93	3.83
	PLX 40 kHz	−76.72	−74.97	−68.40		−82.05	−72.99	−62.08	−67.14	−66.99	−68.47	−57.29	−66.25	−57.51	−54.61	−50.01	−46.58	−68.47	−64.90			−66.73	−73.62	−75.98		−73.69	−72.55	−78.68	−86.70
	PLX CV 40 kHz	−4.16	−4.62	−10.06		−5.90	−11.22	−6.37	−4.09	−6.24	−9.00	−7.35	−10.40	−8.98	−6.53	−6.35	−9.23	0.00	−5.88			−9.06	−5.99	−5.89		−5.01	−7.54	−9.77	−8.65
9	PLR 128 kHz	681.66	672.49	671.12	601.88	725.63	728.27	716.90	769.62	706.79	702.59	700.91	721.82	635.14	619.21	670.28		689.59	690.11	661.45	689.47	684.48	708.39		749.52	690.18	657.97	617.27	650.17
	PLR CV 128 kHz	3.05	1.41	4.89	2.12	2.33	1.46	3.38	3.03	0.46	1.54	3.89	2.28	2.88	4.70	1.27		1.63	1.61	2.64	1.73	2.87	4.81		3.32	3.33	4.34	3.22	3.21
	PLX 40 kHz	−51.18	−55.14	−58.70	−60.36	−58.91	−64.38	−60.60	−69.92	−58.12	−67.84	−73.49	−68.67	−52.47	−54.95	−60.54		−59.18	−66.06	−59.35	−65.67	−53.99	−56.85		−62.34	−78.82	−73.49	−62.08	−66.37
	PLX CV 40 kHz	−7.45	−4.57	−5.46	−4.44	−4.81	−4.82	−6.12	−7.94	−5.59	−5.46	−5.75	−7.44	−5.30	−9.05	−4.06		−4.80	−3.75	−7.95	−4.58	−10.50	−8.55		−6.49	−7.92	−7.11	−8.58	−3.79
10	PLR 128 kHz	311.68	375.30	375.08	385.45		339.77	333.27	427.35	338.58	347.17	314.17						404.26		262.63							298.11	272.84	
	PLR CV 128 kHz	4.47	3.16	2.19	15.60		2.76	2.62	16.73	2.21	1.74	15.12						6.77		9.05							18.17	2.58	
	PLX 40 kHz	−36.09	−39.37	−42.12	−69.74		−45.93	−43.97	−103.98	−31.50	−37.15	−58.79						−84.30		−59.70							−97.08	−41.66	
	PLX CV 40 kHz	−12.86	0.00	−7.74	−97.48		−5.09	−7.80	−84.35	−9.32	−10.17	−82.49						−34.81		−17.52							−106.48	−7.60	
11	PLR 128 kHz	378.22	386.23	365.69	388.59	440.38	432.97	400.58	398.63	400.21	380.80	379.00	367.55	375.81	331.80	337.19	329.98	379.12	379.81	393.02	392.40					367.22	344.44	338.65	369.36
	PLR CV 128 kHz	3.45	3.00	2.83	1.34	2.68	4.25	2.73	2.23	3.82	2.01	2.20	1.55	3.67	4.23	4.84	3.56	2.37	2.40	3.15	1.54					2.87	3.18	4.45	2.21
	PLX 40 kHz	−29.13	−30.66	−28.89	−28.63	−37.04	−36.84	−32.54	−29.75	−39.70	−32.05	−29.78	−21.75	−27.74	−23.59	−23.48	−26.36	−36.59	−42.00	−44.31	−41.43					−31.68	−28.63	−29.84	−32.93
	PLX CV 40 kHz	−6.33	−10.63	−4.63	0.00	−7.49	−10.22	−11.03	−8.82	−13.32	−16.73	−17.01	−6.45	−9.12	−13.91	−13.82	−14.16	−6.27	−8.75	−6.39	−7.17					−11.31	0.00	−15.00	−11.91
12	PLR 128 kHz	516.00	529.17	503.17	487.92	498.46	482.94	508.44		566.15	522.33	536.03	522.37	445.39	407.03	367.36	402.56	527.98	491.78	493.44	486.76	516.46	548.58	537.70	501.07	463.29	462.88	470.15	480.39
	PLR CV 128 kHz	5.38	4.44	3.79	10.58	6.55	7.62	2.59		5.17	3.84	3.91	2.61	4.60	12.40	13.77	10.98	3.79	4.85	5.75	3.08	14.49	6.02	4.11	7.54	4.17	2.71	2.94	2.50
	PLX 40 kHz	−83.77	−91.45	−79.12	−104.47	−92.26	−120.50	−87.33		−99.56	−96.53	−98.54	−84.65	−77.53	−72.08	−78.74	−74.13	−86.15	−98.15	−90.91	−88.69	−101.29	−96.96	−95.30	−101.56	−86.69	−78.55	−78.07	−81.79
	PLX CV 40 kHz	−6.40	−5.07	−10.40	−15.67	−6.50	−52.49	−9.90		−5.83	−4.59	−9.07	−5.71	−6.22	−12.19	−22.98	−8.17	−6.84	−9.41	−7.78	−13.54	−12.62	−6.50	−8.33	−32.38	−14.96	−4.63	−10.02	−4.37
13	PLR 128 kHz	548.15	538.49	522.38						571.77	567.88			551.56	564.13	575.49		599.39	612.28	590.26	598.05	551.40	517.44	512.08	520.30	569.08	583.53	560.57	563.50
	PLR CV 128 kHz	1.28	2.24	5.20						1.43	1.61			1.85	1.36	0.82		1.61	0.85	4.56	1.24	0.84	4.36	1.36	1.59	3.32	2.25	3.41	1.89
	PLX 40 kHz	−76.24	−74.13	−71.29						−75.78	−78.83			−65.99	−74.99	−76.68		−78.05	−82.49	−77.39	−74.99	−73.67	−66.45	−68.35	−68.57	−71.17	−78.31	−70.42	−67.49
	PLX CV 40 kHz	−4.30	−3.26	−6.81						−8.30	−4.78			−6.91	0.00	−4.12		−5.13	0.00	−6.19	−2.95	−3.94	−9.52	−3.54	−4.73	−9.15	−4.79	−8.16	−8.08
14	PLR 128 kHz	485.91	500.53	515.43		479.29	482.05			523.90	489.07	522.82		518.85	522.49			463.08	460.32	451.14		498.58	506.68	511.58	508.69				
	PLR CV 128 kHz	2.95	3.82	2.72		1.75	4.06			4.21	7.25	6.26		4.58	2.67			4.65	3.45	2.39		17.08	2.44	1.71	1.61				
	PLX 40 kHz	−51.81	−52.14	−51.83		−48.69	−48.69			−52.35	−59.23	−53.80		−48.99	−46.51			−45.27	−42.54	−43.36		−54.57	−51.55	−51.58	−48.13				
	PLX CV 40 kHz	−6.66	−6.47	−9.40		−4.69	−6.90			−6.92	−12.99	−13.99		−12.24	−6.07			−12.28	−6.61	−7.59		−9.14	−6.73	−6.68	−2.44				
15	PLR 128 kHz	635.62	659.21	652.73		633.52	612.52	615.93		622.87	631.99	617.43	625.79	690.29	688.98	690.79	687.30	639.11	636.12	688.14		651.25	628.58	617.13	627.84	694.55	699.34	698.65	683.85
	PLR CV 128 kHz	1.23	2.38	2.90		1.83	2.03	1.97		1.31	1.73	0.99	1.27	0.43	2.42	1.98	3.93	1.02	3.42	1.53		2.24	2.11	1.89	1.90	0.88	2.89	2.37	2.23
	PLX 40 kHz	−55.95	−59.86	−57.01		−59.48	−61.04	−68.44		−62.82	−63.43	−58.99	−53.47	−60.37	−66.61	−69.07	−64.09	−55.45	−60.37	−65.30		−54.68	−50.58	−47.18	−50.57	−58.73	−59.25	−57.85	−56.07
	PLX CV 40 kHz	−5.93	−3.63	−7.15		−18.66	−13.53	−8.10		−5.59	−4.92	−1.90	−4.44	−4.86	−7.09	−4.77	−8.61	−6.04	−7.32	−2.16		−7.82	−5.92	−5.49	−5.98	−2.50	−3.68	−4.46	−5.88
16	PLR 128 kHz	998.40	929.63	836.04	848.53	867.11	832.88	853.67	902.33	857.72	818.09	740.02		942.82	900.68	839.25	803.55	808.50	738.97	724.58	706.71								
	PLR CV 128 kHz	2.29	3.53	2.23	2.44	18.66	3.42	3.80	4.72	19.17	4.46	5.32		2.58	3.83	3.59	1.75	6.06	5.57	3.50	2.60								
	PLX 40 kHz	−67.02	−58.92	−51.72	−51.25	−70.64	−56.35	−60.99	−79.28	−103.04	−65.27	−40.83		−68.10	−60.93	−51.15	−50.83	−59.53	−58.13	−61.86	−68.58								
	PLX CV 40 kHz	−7.86	−5.66	−4.44	−5.39	−29.46	−8.07	−8.61	−41.08	−54.50	−22.34	−12.33		−6.57	−8.75	−6.82	−5.23	−6.73	−17.67	−11.96	−8.44								
17	PLR 128 kHz	708.90	813.97	844.32	827.62	666.91	675.40	657.94	635.34	691.23	721.98	743.35	714.29	670.35	704.97	709.82		698.70	664.42	709.72		634.20	642.15	651.08	650.54	642.38	634.14	602.31	
	PLR CV 128 kHz	6.34	3.62	2.66	1.56	3.84	2.86	4.28	2.57	5.22	3.37	2.70	0.96	4.13	2.98	2.64		2.92	2.39	2.47		4.68	1.91	1.49	2.21	3.44	2.46	2.32	
	PLX 40 kHz	−78.04	−97.73	−103.32	−93.08	−91.61	−93.96	−84.87	−80.75	−72.55	−81.32	−96.03	−79.40	−67.43	−74.00	−73.28		−90.67	−100.10	−108.49		−62.77	−53.39	−50.17	−50.47	−50.98	−41.40	−38.99	
	PLX CV 40 kHz	−8.04	−4.42	−5.08	−3.65	−35.50	−15.62	−8.02	−3.40	−2.45	−9.94	−9.97	−2.35	−7.45	−5.88	−5.78		−4.88	−8.61	−6.17		−17.30	−7.77	−3.04	−5.99	−11.40	−7.37	−3.66	
18	PLR 128 kHz		443.72	466.70		436.87	464.45	480.81		422.68	468.13	482.75						423.31	404.96			400.26	451.28	493.58		405.73	418.74	434.19	
	PLR CV 128 kHz		4.33	3.62		1.89	3.48	4.16		3.83	3.65	3.61						2.88	5.01			4.52	6.94	3.33		4.86	2.18	4.02	
	PLX 40 kHz		−62.28	−68.41		−44.55	−49.75	−57.02		−41.56	−51.13	−52.49						−35.21	−34.47			−32.81	−37.24	−32.81		−33.03	−35.57	−39.72	
	PLX CV 40 kHz		−19.20	−7.44		−6.17	−6.57	−6.16		−7.77	−5.24	−2.55						−9.09	−8.32			0.00	−9.18	−7.84		−3.63	−9.23	−7.01	
19	PLR 128 kHz	508.96	512.63	501.27	511.85	466.47	477.71	446.30		456.33	491.49	492.38		506.95	510.99	513.98		503.22	518.33	560.60		523.79	472.48	441.54		484.03	473.55	480.85	483.78
	PLR CV 128 kHz	3.11	2.51	2.44	2.56	3.16	2.98	3.03		4.65	7.09	3.37		2.70	5.17	3.47		3.57	2.55	3.77		3.57	3.42	2.33		3.72	2.90	3.60	2.98
	PLX 40 kHz	−66.19	−62.96	−63.78	−67.33	−65.52	−63.20	−54.04		−61.32	−71.15	−72.07		−65.05	−67.16	−62.08		−70.59	−81.86	−85.51		−69.47	−68.51	−59.87		−59.52	−59.12	−63.29	−63.36
	PLX CV 40 kHz	−5.97	−4.36	−5.02	−5.75	−6.32	−9.39	−8.24		−9.30	−34.81	−7.43		−6.21	−7.14	−10.60		−5.49	−4.92	−5.56		−4.06	−6.88	−5.70		−5.53	−5.61	−5.95	−4.74
20	PLR 128 kHz	489.43	473.36	395.82						391.44	402.95	405.45	399.84	427.86	432.65	436.65	468.98	488.75	486.04	482.94	433.18	431.28	425.45	434.11	428.50	406.09	404.19	385.03	403.78
	PLR CV 128 kHz	1.72	3.45	11.58						1.90	1.72	2.38	2.51	1.83	3.45	2.92	2.57	2.35	8.14	2.52	4.47	1.59	4.60	2.10	1.84	2.20	5.44	3.71	4.20
	PLX 40 kHz	−52.29	−40.65	−34.91						−49.98	−46.98	−47.68	−48.35	−43.93	−50.44	−43.52	−49.99	−64.03	−52.11	−42.71	−46.11	−48.32	−48.51	−45.12	−44.74	−43.78	−43.70	−45.43	−35.92
	PLX CV 40 kHz	−9.93	−12.53	−12.30						−14.70	−14.37	−15.57	−10.26	−5.66	−15.16	−12.49	−11.93	−6.03	−16.19	−11.94	−14.04	−7.41	−14.11	−12.79	−7.03	−7.06	−12.57	−8.76	−16.19

TABLE I. Longitudinal PLR and PLX mean and standard deviation data at 128 kHz and 40 kHz used in the MLM analysis

		Transverse Electrode																											
Participant	Metric	Datapoint																											
		1	2	3	4	5	6	7	8	9	10	11	12	13	14	15	16	17	18	19	20	21	22	23	24	25	26	27	28
1	PLR 128 kHz	276.49	284.88	274.44	207.33					300.24	265.12	217.45		209.21	167.80	176.01		261.67								324.50	269.20	224.24	
	PLR CV 128 kHz	8.52	5.98	8.03	33.24					14.82	8.47	7.25		36.40	38.30	40.04		18.85								3.51	16.30	16.96	
	PLX 40 kHz	−24.16	−24.90	−28.06	−49.47					−61.97	−79.01	−141.62		−51.35	−51.37	−54.74		−45.07								−36.47	−69.82	−106.28	
	PLX CV 40 kHz	−15.33	−16.80	−37.33	−61.04					−30.94	−31.33	−20.78		−24.58	−35.56	−38.21		−49.21								−10.58	−58.10	−49.92	
2	PLR 128 kHz	501.86	489.94		502.05	487.11	472.80	477.07	479.92	501.43	495.27	491.57	490.07	605.27	593.67	630.56	668.38	594.89	556.24	549.80	593.37	617.00	553.23	510.39		520.97	518.88	509.10	517.26
	PLR CV 128 kHz	6.79	4.46		4.02	5.87	6.11	3.29	3.09	4.65	3.41	3.11	4.49	3.69	3.97	4.73	4.82	18.20	5.84	4.20	3.84	8.21	5.26	7.39		4.35	3.64	4.43	3.58
	PLX 40 kHz	−23.68	−63.26		−18.62	−21.10	−18.79	−18.53	−16.85	−26.88	−26.65	−25.29	−21.27	−49.30	−42.67	−41.12	−41.99	−95.27	−36.03	−28.37	−33.58	−50.22	−31.21	−23.46		−25.96	−26.19	−23.83	−21.56
	PLX CV 40 kHz	−49.83	−42.15		−8.68	−29.56	−14.88	−66.33	−64.21	−12.23	−12.14	−15.83	−15.63	−7.78	−8.04	−10.69	−14.75	−87.98	−80.07	−24.31	−23.88	−33.56	−19.35	−24.93		−13.34	−10.87	−18.59	−14.23
3	PLR 128 kHz	373.70	428.70	369.70	341.61	402.35	368.04	352.46	331.28	290.13	272.73	250.73	231.75	239.40	203.33	205.19	195.50	227.47	230.57	238.00	216.17	228.65	227.08		219.21				
	PLR CV 128 kHz	14.90	5.23	7.89	16.76	5.00	2.93	4.26	2.80	5.46	8.02	13.83	5.61	7.54	9.12	11.24	16.97	3.59	5.71	8.66	7.78	7.55	7.21		18.08				
	PLX 40 kHz	−20.00	−32.81	−18.96	−25.45	−23.17	−17.50	−17.22	−17.26	−23.14	−19.36	−16.54	−17.30	−33.40	−17.01	−16.27	−19.52	−10.28	−14.54	−23.08	−8.75	−15.84	−9.72		−14.58				
	PLX CV 40 kHz	−20.13	−85.13	−28.24	−81.42	−21.34	−10.66	−18.18	−8.33	−20.82	−20.06	−16.64	−13.15	−55.95	−48.34	−69.01	−179.23	−32.75	−30.61	−197.85	0.00	−53.41	−28.60		−41.16				
4	PLR 128 kHz	535.12	582.12			458.31	403.51	391.82	382.11	464.59	462.24	472.07		507.05	432.42	383.02	366.52	401.10	412.77	393.40	421.42	424.66	447.04	409.36	390.22	477.29	449.02	469.29	495.15
	PLR CV 128 kHz	4.36	3.45			7.14	3.53	5.84	6.39	2.60	4.57	5.17		5.55	10.65	5.62	3.08	0.67	4.39	4.78	5.38	3.52	2.09	6.03	4.52	5.48	5.15	5.77	5.09
	PLX 40 kHz	−10.37	−20.41			−10.41	−7.08	−6.68	−6.06	−12.34	−11.04	−14.96		−18.17	−14.57	−11.12	−13.36	−12.11	−12.11	−9.50	−10.55	−12.11	−12.11	−10.09	−2.41	−18.47	−14.49	−18.42	−39.12
	PLX CV 40 kHz	−28.55	−34.63			−34.33	−32.29	−32.61	0.00	−9.45	−22.84	−20.42		0.00	−32.94	−20.33	−18.55	0.00	0.00	−31.79	−54.50	0.00	0.00	−37.37	−124.03	−24.57	−25.29	−24.41	−32.92
5	PLR 128 kHz	506.64	505.13			833.76	829.17	829.14	821.29	604.84	598.28			539.59	506.53	531.54	551.51	601.38	576.71	580.75	546.18	482.96	555.32	559.58	578.25	528.56	515.86	534.24	545.81
	PLR CV 128 kHz	2.86	3.77			2.87	3.54	2.60	2.70	2.69	3.78			2.92	10.58	5.89	2.63	4.62	2.90	2.53	4.50	7.39	2.11	1.87	3.11	3.02	2.83	3.06	2.92
	PLX 40 kHz	−18.88	−20.11			−58.01	−55.09	−59.91	−61.55	−25.15	−23.52			−22.21	−19.56	−15.72	−15.88	−16.51	−12.30	−14.58	−13.24	−8.75	−12.92	−13.49	−13.67	−15.82	−14.10	−14.17	−13.65
	PLX CV 40 kHz	−10.65	−23.46			−22.72	−10.76	−4.81	−4.35	−9.48	−8.35			−13.77	−37.63	−23.09	−19.67	−17.49	−8.57	−20.59	−17.91	−34.64	−18.24	−19.03	−19.52	−18.85	−20.26	−20.41	−19.44
6	PLR 128 kHz	683.39	679.17	464.43	440.46	649.86				647.60	683.24	725.50	712.77	674.21	735.09	701.27	651.46												
	PLR CV 128 kHz	4.08	11.03	7.87	6.79	21.49				2.22	1.97	3.48	6.52	5.22	3.42	8.54	12.02												
	PLX 40 kHz	−40.53	−51.62	−40.44	−26.09	−68.73				−29.29	−33.75	−33.76	−31.37	−43.99	−51.85	−126.31	−145.10												
	PLX CV 40 kHz	−11.28	−68.41	−69.34	−61.79	−87.18				−7.72	−10.19	−10.56	−11.71	−15.06	−25.93	−44.43	−43.92												
7	PLR 128 kHz	295.21				325.34	259.55			394.28	380.12	352.18		287.92	253.90	308.37	323.94	376.79	448.84	434.42	341.97	310.89	255.71	276.08	272.97	323.42	299.83	282.15	
	PLR CV 128 kHz	9.80				10.49	6.48			4.17	5.77	7.74		16.78	7.31	11.90	14.69	2.23	18.90	24.26	9.49	12.05	15.26	11.19	8.78	5.66	10.32	11.92	
	PLX 40 kHz	−11.57				−9.97	−10.69			−10.13	−11.12	−27.81		−32.61	−26.25	−19.46	−16.81	−41.99	−146.31	−118.99	−27.90	−19.33	−14.27	−13.12	−12.25	−39.08	−28.25	−18.44	
	PLX CV 40 kHz	−45.31				−73.12	−41.43			−56.51	−43.44	−162.24		−49.60	−38.70	−22.22	−27.89	−40.31	−100.41	−124.87	−87.94	−52.78	−35.95	−37.73	−49.94	−22.75	−23.12	−38.05	
8	PLR 128 kHz	350.29	370.65	315.49		325.64	310.71	289.60	288.04	257.28	274.58	295.95	331.73	310.72	348.79	305.77	304.24		247.28			289.30	291.69	304.91		287.27	293.81	306.57	299.18
	PLR CV 128 kHz	12.94	23.06	41.20		11.53	7.08	5.94	6.73	22.86	12.67	12.07	6.91	4.69	6.16	4.42	7.56		3.44			8.68	5.32	5.49		5.27	6.69	6.19	5.61
	PLX 40 kHz	−107.68	−116.56	−32.13		−22.48	−23.00	−20.43	−17.83	−41.97	−18.68	−26.32	−25.39	−16.66	−17.36	−11.57	−8.94		−0.26			−13.96	−8.64	−4.43		−15.86	−21.15	−18.45	−17.15
	PLX CV 40 kHz	−99.02	−60.12	−38.51		−54.59	−42.92	−53.34	−36.26	−80.04	−96.88	−74.14	−89.50	−28.60	−34.57	−15.06	−36.30		−479.58			−20.15	−40.76	−80.99		−25.55	−16.35	−25.43	−15.78
9	PLR 128 kHz	479.19	434.09	430.03		473.13	474.07	453.11	472.88	410.57	420.03	413.57	421.73	488.62	483.04	501.89		366.95	368.09	367.50	370.21	357.28	357.54	387.13		354.19	335.21	323.94	336.05
	PLR CV 128 kHz	7.61	2.98	5.18		3.17	2.49	2.47	2.09	3.62	2.13	2.59	4.76	4.79	6.56	3.16		5.42	3.49	4.75	5.70	4.84	3.57	4.06		8.54	3.31	6.10	3.45
	PLX 40 kHz	−33.07	−19.43	−18.92		−18.16	−16.65	−16.00	−19.24	−20.62	−19.46	−21.12	−21.85	−30.03	−32.07	−34.41		−19.12	−17.11	−20.80	−18.36	−18.84	−20.22	−21.35		−36.63	−28.29	−30.39	−30.93
	PLX CV 40 kHz	−17.73	−17.25	−29.75		−13.11	−16.03	−20.28	−16.13	−15.75	−14.47	−11.57	−22.09	−14.01	−16.37	−12.77		−29.68	−6.71	−18.75	−20.39	−24.88	−14.40	−15.26		−126.31	−69.57	−59.34	−10.50
10	PLR 128 kHz									114.87	137.95											173.94	210.18						
	PLR CV 128 kHz									5.26	7.65											15.68	13.93						
	PLX 40 kHz									−17.60	−22.93											−21.34	−36.98						
	PLX CV 40 kHz									−11.77	−36.90											−13.53	−54.09						
11	PLR 128 kHz		262.47	260.94	263.32	350.75	364.23	358.19	349.32	335.13	311.95	274.44	276.42	249.64	236.22	246.64	245.65	233.30	266.75	283.26	278.74	277.83	248.88	278.90	305.85	360.77	379.20	395.47	413.74
	PLR CV 128 kHz		4.35	4.95	4.42	5.77	7.72	4.69	5.57	5.54	9.11	6.14	4.15	8.73	9.08	8.90	11.09	6.50	8.96	7.59	5.56	4.63	6.71	12.86	8.42	4.64	5.64	8.06	3.09
	PLX 40 kHz		−33.56	−24.67	−32.72	−27.70	−20.95	−19.24	−17.50	−15.62	−14.94	−18.41	−14.59	−10.74	−8.88	−8.16	−11.35	−11.67	−14.05	−14.32	−14.32	−16.55	−14.83	−14.58	−14.32	−24.87	−23.45	−27.43	−28.63
	PLX CV 40 kHz		−34.58	−16.07	−11.23	−11.83	−14.84	−17.34	−20.56	−22.97	−13.61	−19.42	−9.62	−35.63	−34.58	−30.73	−31.46	−30.20	−9.62	0.00	0.00	−20.70	−14.07	−9.36	0.00	−14.55	−13.74	−13.00	−17.68
12	PLR 128 kHz	308.16	327.43			341.21		442.94		414.57	311.29	420.64	380.58	256.03	343.61	421.18	426.40					335.74	325.54	458.50	480.68	370.86	344.18	371.89	362.92
	PLR CV 128 kHz	7.97	7.09			21.50		2.76		15.34	16.11	18.47	3.21	38.21	23.17	4.31	4.45					7.87	24.89	8.30	4.57	4.14	7.73	11.15	3.99
	PLX 40 kHz	−50.55	−46.91			−74.87		−29.25		−43.96	−62.65	−30.24	−18.15	−67.18	−51.22	−45.88	−41.31					−83.11	−44.15	−35.71	−36.41	−73.88	−57.57	−59.61	−61.89
	PLX CV 40 kHz	−39.39	−17.14			−98.43		−22.88		−39.19	−42.06	−49.91	−20.09	−36.55	−16.90	−9.81	−15.04					−105.56	−22.03	−14.17	−11.45	−9.28	−12.35	−15.01	−11.92
13	PLR 128 kHz	278.32	308.90	361.85						258.48	257.44			298.81	323.50	324.66		301.52	300.85	326.26	309.00	276.43	269.53	269.97	275.40	334.81	349.12	391.11	322.12
	PLR CV 128 kHz	1.63	10.29	21.11						4.25	1.85			2.56	6.28	4.71		7.56	1.60	13.06	1.76	1.43	9.73	1.52	9.38	7.18	12.48	20.71	6.53
	PLX 40 kHz	−14.32	−10.19	−12.88						−8.07	−8.27			−27.72	−31.97	−31.40		−21.40	−21.47	−19.63	−14.32	−18.11	−19.36	−20.86	−23.51	−21.20	−20.45	−19.77	−11.86
	PLX CV 40 kHz	0.00	−35.06	−70.27						−29.84	−31.51			−8.75	−12.68	−11.93		−12.24	0.00	−16.01	0.00	−20.03	−20.73	−9.75	−59.86	−6.49	−12.34	−36.04	−56.58
14	PLR 128 kHz	188.98	225.04	237.21						254.39	252.85	253.53			243.78	245.20			222.01	226.89	231.73	247.96	256.35	251.06	245.66	237.20	254.52	271.16	270.54
	PLR CV 128 kHz	8.05	3.47	2.25						5.16	3.10	3.61			3.76	2.25			3.72	2.42	2.47	10.44	3.76	3.12	2.99	4.23	4.13	2.59	1.10
	PLX 40 kHz	−9.09	−6.06	−6.06						−11.09	−11.83	−11.25			−6.30	−6.06			−6.06	−6.06	−6.06	−7.57	−7.66	−6.36	−6.06	−10.36	−10.09	−11.34	−12.11
	PLX CV 40 kHz	−48.74	0.00	0.00						−20.57	−11.00	−19.55			−18.96	0.00			−20.41	0.00	0.00	−35.20	−35.41	−20.93	0.00	−37.58	−28.43	−21.85	0.00
15	PLR 128 kHz	437.69	474.51	513.81		535.35	567.52	551.02		455.28	474.35	482.26	479.84	416.72	438.84	469.97	466.10	437.31	444.92	482.73		494.65	527.42	505.64	530.20	479.71	515.02	545.76	528.50
	PLR CV 128 kHz	4.90	2.63	4.79		3.95	2.92	9.22		3.48	1.60	1.80	0.88	0.65	3.47	1.74	2.16	1.94	2.70	2.29		2.58	4.01	2.98	3.18	0.97	4.38	3.76	8.28
	PLX 40 kHz	−27.26	−31.76	−31.05		−30.87	−30.56	−25.27		−25.24	−27.59	−29.58	−28.04	−13.33	−17.92	−21.04	−19.58	−17.87	−12.58	−12.21		−29.78	−33.34	−36.34	−36.34	−23.92	−26.77	−28.37	−27.31
	PLX CV 40 kHz	−12.39	−9.32	−7.65		−7.77	−4.17	−13.20		−13.79	−11.01	−6.60	−10.63	−20.33	−6.76	−14.47	−13.22	−7.58	−12.96	−6.25		−5.87	−9.13	0.00	0.00	−5.66	−11.22	−10.06	−13.41
16	PLR 128 kHz	602.99	555.38	536.65	545.93			719.81	480.70					685.39	678.99	667.22	660.00									593.55	529.07	462.52	482.62
	PLR CV 128 kHz	5.46	3.79	3.33	3.23			12.65	15.49					8.46	3.63	3.47	3.37									5.86	10.60	15.45	13.47
	PLX 40 kHz	−31.99	−31.74	−27.98	−29.69			−63.36	−103.04					−46.37	−39.69	−38.22	−33.59									−43.02	−52.01	−44.40	−36.20
	PLX CV 40 kHz	−10.41	−25.20	−9.37	−7.77			−36.87	−22.97					−45.24	−6.62	−7.79	−6.62									−44.47	−56.41	−43.87	−38.99
17	PLR 128 kHz	574.15	636.12	700.07	654.95	504.86	519.37	503.78	484.69	461.75	502.00	552.84	543.90	487.92	535.67	547.96		513.51	537.57	573.92		436.22	456.02	492.05	488.16	406.12	468.85	483.37	
	PLR CV 128 kHz	4.46	3.96	5.04	6.65	1.82	1.83	2.47	1.76	3.01	4.38	4.13	1.89	4.76	4.42	3.95		4.92	2.51	2.58		2.18	2.61	1.79	2.26	7.78	1.84	2.32	
	PLX 40 kHz	−27.65	−35.38	−50.20	−45.56	−21.26	−22.30	−19.58	−17.27	−21.93	−22.04	−31.73	−39.04	−29.25	−38.00	−36.97		−18.02	−24.81	−35.13		−15.97	−16.87	−18.48	−21.92	−6.80	−13.90	−21.03	
	PLX CV 40 kHz	−5.74	−13.39	−16.19	−19.17	−11.05	−4.64	−14.48	−8.54	−8.11	−7.02	−19.16	−3.49	−15.92	−8.14	−9.59		−23.25	−11.20	−14.37		−13.04	0.00	−13.85	−7.89	−54.92	−24.63	−11.82	
18	PLR 128 kHz		263.58	301.71		190.32	192.06	192.01		227.64	238.13	250.76		259.64	216.38	246.26			236.22			191.40	196.40	198.76		189.38	199.24	211.03	
	PLR CV 128 kHz		9.81	5.02		6.03	4.61	4.79		4.46	4.87	4.15		25.99	19.13	4.54			7.56			2.83	3.59	4.71		16.24	4.78	3.75	
	PLX 40 kHz		−16.96	−21.12		−8.29	−9.25	−10.79		−9.09	−6.14	−6.06		−77.93	−57.65	−24.23			−11.86			−6.06	−6.06	−6.51		−18.37	−8.45	−12.56	
	PLX CV 40 kHz		−21.61	−14.42		−36.22	−32.99	−23.32		−34.82	−11.55	0.00		−189.71	−87.49	−112.95			−126.01			0.00	0.00	−24.85		−146.28	−35.52	−44.84	
19	PLR 128 kHz	175.65	170.78	170.07	171.48	297.85	286.62	268.39		287.13	326.97	331.96		289.15	282.68	291.87						270.04	265.17	241.81		181.24	183.35	197.89	212.99
	PLR CV 128 kHz	5.97	3.14	3.23	3.16	3.68	4.37	4.56		5.03	6.14	19.36		4.78	4.40	3.65						2.35	4.45	5.46		1.65	3.38	4.71	3.72
	PLX 40 kHz	−16.15	−17.91	−18.09	−17.57	−45.85	−30.13	−24.81		−36.43	−41.88	−47.07		−42.78	−41.24	−38.61						−31.80	−29.90	−24.69		−23.76	−22.10	−23.62	−23.78
	PLX CV 40 kHz	−21.65	−6.97	−3.84	−10.40	−11.38	−13.74	−14.42		−10.89	−8.26	−6.44		−12.88	−6.77	−69.89						−10.76	−15.97	−6.62		−7.07	−13.93	−10.83	−11.99
20	PLR 128 kHz	299.54	303.37	241.81						252.87	245.64	219.82	233.69	241.59	255.35	271.22	280.26	268.79	270.86	274.13	248.76	253.72	249.71	245.83	237.97	237.23	249.14	262.97	230.75
	PLR CV 128 kHz	7.06	6.47	11.56						7.60	10.02	8.70	7.12	7.46	8.92	3.68	3.63	1.50	5.06	4.42	6.19	0.00	7.12	8.16	4.65	7.25	7.50	10.55	7.07
	PLX 40 kHz	−21.20	−19.09	−19.51						−8.75	−8.75	−11.48	−11.05	−11.93	−9.16	−11.30	−17.21	−17.50	−14.64	−8.89	−8.75	−8.75	−8.75	−8.75	−8.75	−8.75	−11.90	−13.46	−7.29
	PLX CV 40 kHz	−20.56	−20.67	−23.72						0.00	0.00	−36.47	−35.33	−35.68	−20.36	−35.95	−9.20	0.00	−28.31	−12.70	0.00	0.00	0.00	0.00	0.00	0.00	−43.54	−32.72	−57.17

TABLE II. Transverse PLR and PLX mean and standard deviation data at 128 kHz and 40 kHz used in the MLM analysis
